# The Conserved Function of Skp1 in Meiosis

**DOI:** 10.3389/fgene.2012.00179

**Published:** 2012-09-14

**Authors:** Josh D. McLoud, Ming Yang

**Affiliations:** ^1^Department of Botany, Oklahoma State UniversityStillwater, OK, USA

Many components of regulatory networks governing basic cellular functions are highly homologous in diverse species. An important question is how such conservation of individual components is translated into the conservation at the level of regulatory networks. An intriguing case regarding this question is concerned with the function of the Skp1 protein in meiosis. Skp1 is a conserved subunit of the Skp1-Cullin-F-box-protein (SCF) E3 ubiquitin ligase (Tyers and Jorgensen, 2000; Willems et al., 2004). For nearly 13 years, a distinct meiotic phenotype was described only in a null mutant of Skp1 in *Arabidopsis thaliana*, namely *arabidopsis-skp1-like1-1* (*ask1-1*) (Yang et al., 1999; Yang and Ma, 2001; Wang et al., 2004; Wang and Yang, 2006). It raises the question of whether such a phenotype indicates a plant-specific function of Skp1 (or the corresponding SCF) in meiosis. However, a strikingly similar meiotic phenotype was recently reported by Okamoto et al. (2012) in *skp1* mutants of fission yeast (*Schizosaccharomyces pombe*). These phenotypic similarities suggest the presence of a conserved network involving an SCF that regulates the progression of recombination in meiosis.

The first similarity is that the numbers of spores produced by a meiocyte range from two to more than four spores in *ask1-1* (Yang et al., 1999) and the yeast *skp1* mutants. In many meiotic mutants, the meiotic products produced by a meiocyte are either more or fewer than the normal four spores. The wide range of meiotic product number per meiocyte places *ask1-1* and the yeast *skp1* mutants in a rare group. The second similarity is indicated by the morphology and behavior of chromosomes during meiosis I. Chromosomes entangle, non-disjoin, are stretched by the anaphase spindle, and as a result, are unevenly distributed to meiotic products in both *ask1-1* (Yang et al., 1999; Wang et al., 2004) and the yeast *skp1* mutants. Chromosomes are asynaptic in prophase I in *ask1-1* (Wang et al., 2004). Fission yeast normally undergoes homologous chromosome pairing instead of synapsis in prophase I. It is unknown whether the homologous chromosome pairing in the yeast *skp1* mutants is also affected, but it may be so based on the overall similarities in chromosome behavior between *ask1-1* and the yeast *skp1* mutants. Spindle behavior is intimately linked to chromosome behavior. It is unsurprising that the spindle seems similarly affected by chromosome entanglement in *ask1-1* and the yeast *skp1* mutants. Although the experimental approaches somewhat differ in the investigations of the two organisms – study of still images in *Arabidopsis* and study of time-lapsed videos in yeast, similar conclusions can be made on the impact of entangled chromosomes on the spindle morphology. The *Arabidopsis* work found anaphase spindles that were either shorter than metaphase spindles or longer than typical anaphase spindles in *ask1-1* (Yang and Ma, 2001). The yeast work revealed bending, collapsing, reforming, and re-elongation of spindles. It can be said that spindles are deformed in a similar fashion when they struggle to separate entangled chromosomes in the *skp1* mutants of both organisms. Lastly, the underlying cause of the chromosome entanglement may be attributed to the presence of unresolved recombination intermediates in *ask1-1* and the yeast *skp1* mutants. In *ask1-1*, electron-dense materials abnormally accumulate on chromosomes in prophase I (Wang et al., 2004), the dynamics of a cohesin on meiotic chromosomes is altered (Zhao et al., 2006), and recombination is promoted in plants heterozygous for *ask1-1* (Wang and Yang, 2006). In comparison, several proteins involved in recombination are retained longer than usual on meiotic chromosomes in the yeast *skp1* mutants. Thus, evidence from both *Arabidopsis* and yeast suggests that Skp1 is involved in promoting the resolution of recombination intermediates in meiosis. Perhaps a moderate reduction in the ASK1 function in *Arabidopsis* plants heterozygous for *ask1-1* enhances the formation of recombination intermediates not to a toxic level so that recombination frequency is increased.

The Skp1 protein is not expected to be directly involved in the recombination process, and yet the phenotypes of its *Arabidopsis* and yeast mutants closely resemble each other. It is also noted that the yeast work was carried out without the knowledge of the *Arabidopsis* work (The *Arabidopsis* work was not cited by the yeast paper). The converging evidence from both *Arabidopsis* and yeast suggests the existence of a conserved network in the regulation of recombination progression in meiosis. An F-box protein with a DNA helicase domain is suggested by Okamoto et al. (2012) to be the one linking Skp1 to the recombination process in yeast. The *Arabidopsis* genome does not contain a homolog of the yeast F-box-associated DNA helicase (based on BLAST search using the yeast protein sequence as query) although it has many proteins containing either an F-box or a DNA helicase domain. Further elucidation of the predicted network in diverse species will likely reveal novel aspects in the regulation of meiotic recombination.
